# Prevalence and Associated Risk Factors of *Cyclospora cayetanensis* in Immunocompromised Patients: A Systematic Review and Meta-Analysis

**DOI:** 10.1155/cjid/8837624

**Published:** 2025-08-28

**Authors:** Ali Ghorbani, Rostam Menati, Farshad Kakian, Ali Pouryousef, Asma Mousivand, Kambiz Karimi, Farajolah Maleki, Ali Asghari, Jalil Feizi

**Affiliations:** ^1^Department of Microbiology and Virology, School of Medicine, Kerman University of Medical Sciences, Kerman, Iran; ^2^Psychosocial Injuries Research Center, Ilam University of Medical Sciences, Ilam, Iran; ^3^Department of Nursing, School of Nursing, Larestan University of Medical Sciences, Larestan, Iran; ^4^Leishmaniasis Research Center, Sabzevar University of Medical Sciences, Sabzevar, Iran; ^5^Department of Pathobiology, School of Veterinary Medicine, Shiraz University, Shiraz, Iran; ^6^Department of Medical Parasitology and Mycology, School of Medicine, Shiraz University of Medical Sciences, Shiraz, Iran; ^7^Zoonotic Diseases Research Center, Ilam University of Medical Sciences, Ilam, Iran; ^8^Clinical Research Development Unit, Shahid Mostafa Khomeini Hospital, Ilam University of Medical Sciences, Ilam, Iran; ^9^Medical Microbiology Research Center, Qazvin University of Medical Sciences, Qazvin, Iran; ^10^Department of Internal Medicine, School of Medicine, Ilam University of Medical Sciences, Ilam, Iran

**Keywords:** cancer, *Cyclospora cayetanensis*, hemodialysis, immunocompromised patients, prevalence, transplant

## Abstract

**Background:** *Cyclospora cayetanensis*, an opportunistic protozoan parasite, poses significant risks to immunocompromised patients, including those with cancer, transplants, or on hemodialysis. The aim of this study was to determine the pooled prevalence of *C. cayetanensis* in immunocompromised individuals (cancer, transplant, and hemodialysis) and assess the associated risk factors compared to nonimmunocompromised controls.

**Methods:** A comprehensive search of international databases was conducted for studies published up to October 18, 2024, focusing on cross-sectional and case–control studies that reported *Cyclospora cayetanensis* prevalence in immunocompromised patients. Comprehensive meta-analysis (CMA) software was used to calculate pooled prevalence and odds ratios (ORs), with random-effects models applied to account for heterogeneity based on statistical thresholds. Sensitivity analysis was performed to assess the robustness of pooled prevalence and weighted ORs. Meta-regression analysis was used to evaluate associations between prevalence and variables such as publication year, sample size, and the Human Development Index (HDI). Subgroup analyses were conducted based on geographical regions, publication years, sample sizes, HDI values, income levels, and patient types. This systematic review included 19 studies/22 datasets, comprising 2084 immunocompromised patients and 954 controls across eight countries.

**Results:** The pooled prevalence of *C. cayetanensis* in immunocompromised patients was 4% (95% CI: 2.1%–7.2%), significantly higher than the 1.2% (95% CI: 0.4%–3.4%) in controls, resulting in an OR of 5.4 (95% CI: 2.6–10.8, *p* < 0.001). Transplant recipients exhibited the highest pooled prevalence of *C. cayetanensis* at 5%, indicating the importance of targeted screening and preventive measures for this high-risk group.

**Conclusions:** Despite the relatively low pooled prevalence of *C. cayetanensis* infection in immunocompromised patients, its notable occurrence compared to controls and its opportunistic nature underscore the need for enhanced surveillance and targeted prevention strategies, especially in high-risk populations and regions with higher exposure.

## 1. Introduction

*Cyclospora cayetanensis,* an apicomplexan protozoan, causes cyclosporiasis, a gastrointestinal (GI) illness transmitted through contaminated food and water [1]. Unlike some coccidian parasites, *C. cayetanensis* oocysts require environmental sporulation to become infectious, preventing direct person-to-person transmission and highlighting produce and water contamination as the main infection route [2]. Outbreaks have been linked to fresh fruits and vegetables like berries, cilantro, basil, and lettuce in multiple countries [2, 3].

A recent meta-analysis estimated the global prevalence of *C. cayetanensis* in humans at 3.4%, with rates varying from negligible in high-income countries (0.4%) to higher rates in low-income regions, particularly Africa (5.9%), and underserved populations (over 7%) [4].

Immunocompromised individuals, including those with human immunodeficiency virus (HIV)/acquired immunodeficiency syndrome (AIDS), transplant recipients, cancer patients on chemotherapy, and dialysis patients, face a significantly increased risk of infection [5–8]. Prevalence among HIV-positive individuals has varied widely (0%–48%) [9]. A global systematic review and meta-analysis estimated a pooled prevalence of 3.89% (95% CI: 2.62–5.40) in people living with HIV/AIDS, with diarrhea and low CD4 counts identified as risk factors in immunosuppressed individuals [10].

Cyclosporiasis in immunocompetent individuals typically presents as self-limited diarrhea with abdominal discomfort, nausea, fatigue, low-grade fever, and weight loss, resolving within weeks. Immunocompromised patients, however, often suffer chronic, relapsing, or prolonged diarrhea, leading to malnutrition, dehydration, and increased morbidity. Limited evidence suggests that *C. cayetanensis* can exacerbate conditions like HIV-related GI mucosal damage [10–12].

Despite this, existing data on prevalence and risk factors in high-risk groups remain fragmented, with heterogeneous diagnostic methods (microscopy vs. PCR), small study sizes, and diverse geographic coverage [9]. No comprehensive synthesis has yet integrated data across different immunocompromised populations to assess disease burden, subgroup differences, or regionally relevant trends. Understanding the true prevalence of *C. cayetanensis* in immunosuppressed populations is critically needed: it informs clinical guidelines (screening, diagnosis, prophylaxis), directs public health and food-safety interventions (especially given the environmental route of transmission), and allows identification of the most vulnerable subgroups, such as patients with cancer, transplant recipients, and dialysis patients.

Therefore, this systematic review and meta-analysis aim to synthesize available evidence on the prevalence and risk factors of *C. cayetanensis* infection in immunocompromised populations (those with cancer, organ transplantation, and hemodialysis) and immunocompetent controls. By consolidating existing studies, we aim to clarify the epidemiological patterns and burden in these high-risk groups, addressing important knowledge gaps and supporting more effective health policy and clinical practice.

## 2. Methods

### 2.1. Ethical Considerations

This study involved secondary analysis of data extracted exclusively from the previously published peer-reviewed literature and did not involve direct interaction with human participants. Therefore, informed consent and individual ethical approval were not required. However, the research protocol was reviewed and approved by the Ethics Committee of Qazvin University of Medical Sciences (Approval No. IR.QUMS.REC.1403.498). The ethical approval pertained to the appropriate handling, management, citation, and synthesis of published data in accordance with responsible research practices and academic integrity standards.

### 2.2. Study Design and Search Strategy

This systematic review and meta-analysis was conducted following the PRISMA 2020 guidelines [13]. A comprehensive literature search was carried out to identify all relevant studies reporting the prevalence of *C. cayetanensis* in immunocompromised populations and their immunocompetent controls. The following electronic databases were searched from inception through October 18, 2024: PubMed, Web of Science, Scopus, ScienceDirect, and ProQuest. These databases were selected to maximize the coverage of both biomedical and interdisciplinary literature and to minimize publication bias. In addition, Google Scholar was searched for gray literature. Gray literature includes sources not found in official scientific databases. These sources may include papers, theses, and/or dissertations. The reference lists of all included studies and relevant systematic reviews were manually screened for additional eligible articles. The search strategy combined Medical Subject Headings (MeSH) and free-text keywords related to the target population and the pathogen. The main search terms included (“*Cyclospora cayetanensis*” OR “*Cyclospora*” OR “cyclosporiasis”) AND (“prevalence” OR “frequency” OR “occurrence” OR “epidemiology”) AND (“immunocompromised” OR “immunosuppressed” OR “cancer” OR “transplant” OR “hemodialysis”). Boolean operators (AND/OR), truncation, and database-specific syntax were used to tailor the searches to each platform. No language restrictions were applied. Duplicate records were removed using EndNote (version 7), and titles and abstracts were independently screened by two reviewers.

### 2.3. Eligibility Criteria

This review included cross-sectional and case–control studies of immunocompromised patients (cancer, organ transplant, and hemodialysis) and immunocompetent controls with confirmed *C. cayetanensis* infection or colonization, without time or language restrictions. Excluded were studies focused solely on other immunocompromised groups, general population studies, reviews, editorials, case reports, and studies lacking extractable prevalence data for meta-analysis.

### 2.4. Data Extraction and Quality Assessment

Two independent reviewers screened titles, abstracts, and full texts of potentially eligible articles using a standardized form. Disagreements were resolved through discussion or consultation with a third reviewer. Extracted data included author name, implementation and publication years, country, continent, World Health Organization (WHO) region, Human Development Index (HDI), country income level, sample size, study design, diagnostic method, and patient type. The quality of studies was evaluated using the Joanna Briggs Institute (JBI) critical appraisal checklist for prevalence studies [14]. Scores of > 6 were classified as high quality, 4–6 as moderate quality, and those with a score of ≤ 3 rate as low quality.

### 2.5. Statistical Analysis

A meta-analysis was conducted using comprehensive meta-analysis (CMA) v3 software and a random-effects model to address between-study heterogeneity. Prevalence estimates were presented as proportions with 95% confidence intervals (CIs). Heterogeneity was measured using the *I*^2^ statistic, with values exceeding 50%, indicating substantial heterogeneity. The link between *C. cayetanensis* infection and immunocompromised patients was evaluated using weighted odds ratios (ORs) from case–control studies. Subgroup analyses were performed to identify the prevalence and potential sources of heterogeneity based on publication year, country, continent, WHO region, patient type (cancer, transplant, hemodialysis), HDI value, country income level, and sample size. Income classifications were determined according to the World Bank's country income groups (low, lower-middle, upper-middle, and high income) based on gross national income (GNI) per capita (https://blogs.worldbank.org/en/opendata/world-bank-country-classifications-by-income-level-for-2024-2025). HDI values and categories (low, medium, high, very high) were obtained from the United Nations Development Program (UNDP) Human Development Reports (https://hdr.undp.org/). A sensitivity analysis was conducted by excluding individual studies to reassess the pooled prevalence and weighted OR, confirming the robustness of the findings against potential biases. Meta-regression was employed to assess the potential association between *C. cayetanensis* prevalence in immunocompromised patients and quantitative variables, including publication year, sample size, and HDI value. Publication bias was assessed via funnel plot inspection and Egger's test. A *p*-value of < 0.05 indicated statistical significance for all analyses, with all statistical tests being two-tailed.

## 3. Results

### 3.1. Study Selection and Characteristics

**Figure 1:**
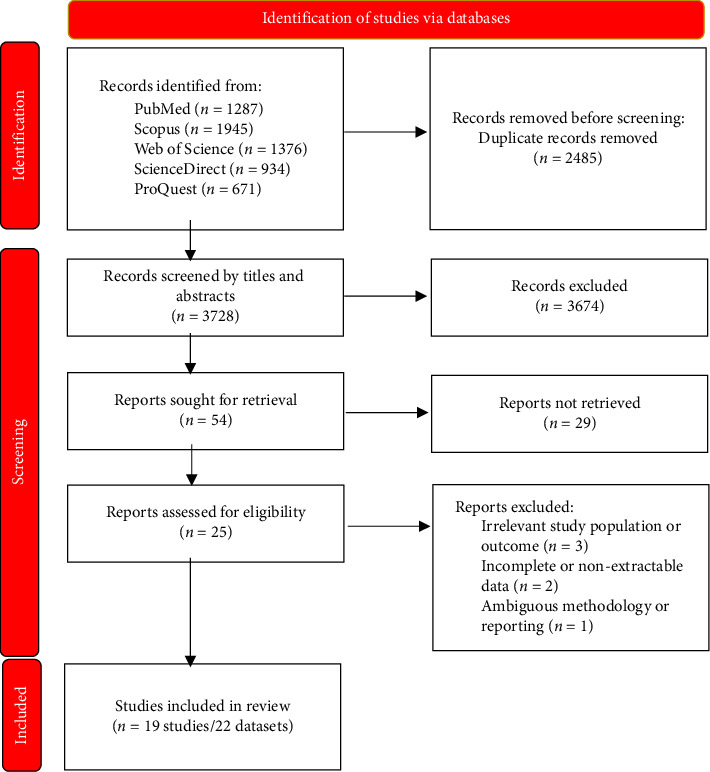
The PRISMA 2020 flow diagram depicting the process of included studies in the present systematic review.

A comprehensive literature search was conducted across five major electronic databases up to October 18, 2024. The number of records identified from each database was as follows: PubMed (*n* = 1287), Scopus (*n* = 1945), Web of Science (*n* = 1376), ScienceDirect (*n* = 934), and ProQuest (*n* = 671), yielding a total of 6213 records. After removing duplicates and screening titles and abstracts, 54 studies were selected for full-text assessment. Of these, 25 studies initially met the eligibility criteria. However, six studies were excluded during data extraction due to the following reasons: irrelevant study population or outcomes (*n* = 3), incomplete or nonextractable data (*n* = 2), and unclear methodology or reporting (*n* = 1). Consequently, 19 studies/22 datasets were included in the final qualitative synthesis and meta-analysis that met the inclusion criteria [5, 7, 8, 15–30] (Figure 1). These studies included 2084 immunocompromised patients and 954 controls across eight countries and four continents. The majority were case–control studies (13 datasets), with the rest being cross-sectional designs (nine datasets). Patient categories included cancer patients (11 datasets, 1099 individuals), organ transplant recipients (seven datasets, 653 individuals), and hemodialysis patients (four datasets, 332 individuals). Diagnostic methods used were microscopic examination (18 datasets) and polymerase chain reaction (PCR) (four datasets) (Table 1). Based on the JBI checklist, nine studies were rated as high quality and 10 studies as moderate quality (Supporting Table 1).

### 3.2. Weighted Prevalence of *C. cayetanensis* in Immunocompromised Patients and Controls

**Figure 2:**
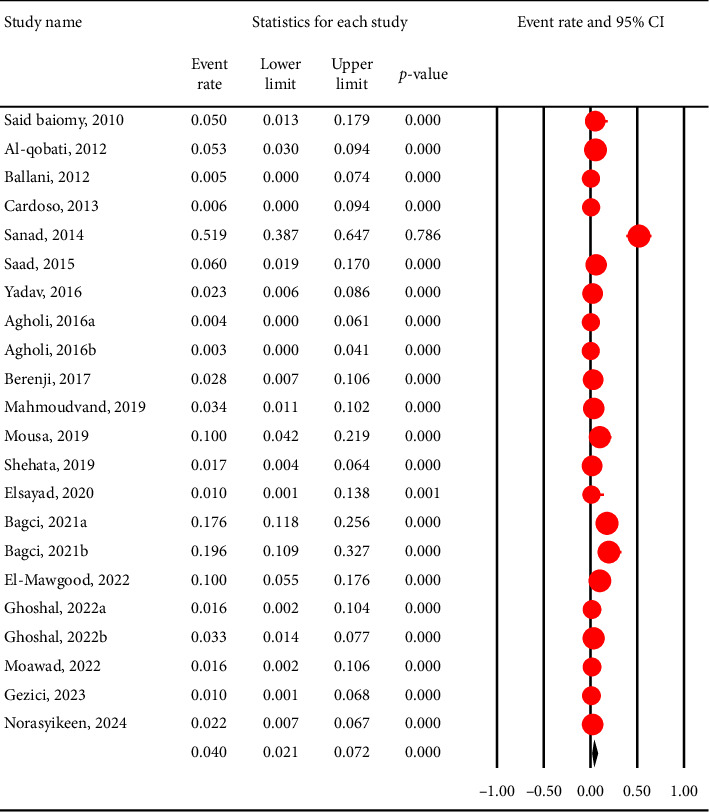
Forest plot showing the prevalence of *C. cayetanensis* infection among immunocompromised patients. Each horizontal black line represents the 95% confidence interval (CI) for the prevalence reported in an individual study. The red circles indicate the point estimate (event rate) for each study, with the size of the circle proportional to the study's weight in the meta-analysis. The vertical solid line at 0 represents the null value (no events), while the vertical dashed line represents the overall pooled prevalence estimated from the random-effects model. The diamond at the bottom summarizes the pooled prevalence and its 95% CI, with the center indicating the point estimate and the horizontal tips representing the CI limits. ^∗^Due to software limitations, studies with 0% prevalence were assigned a minimal value (e.g., 1%) for the analysis. This may lead to a slight overestimation of the pooled prevalence. Readers are advised to consider this methodological limitation when interpreting the pooled prevalence results.

**Figure 3:**
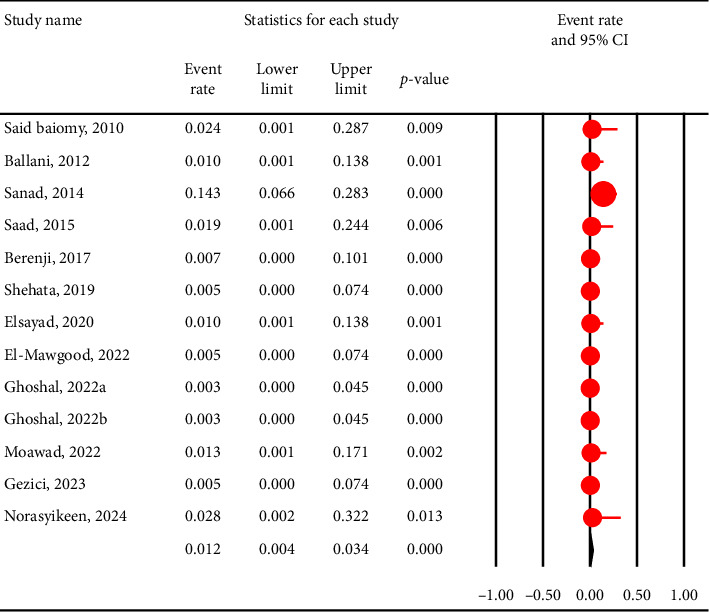
Forest plot showing the prevalence of *C. cayetanensis* infection among nonimmunocompromised controls. Each horizontal black line represents the 95% confidence interval (CI) for the prevalence reported in an individual study. The red circles indicate the point estimate (event rate) for each study, with the size of the circle proportional to the study's weight in the meta-analysis. The vertical solid line at 0 represents the null value (no events), while the vertical dashed line represents the overall pooled prevalence estimated from the random-effects model. The diamond at the bottom summarizes the pooled prevalence and its 95% CI, with the center indicating the point estimate and the horizontal tips representing the CI limits.

**Figure 4:**
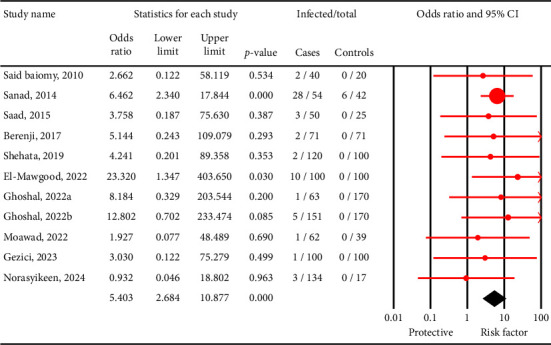
Forest plot showing the weighted random-effects odds ratio (OR) with 95% confidence intervals (CIs) for *C. cayetanensis* infection in immunocompromised patients compared with nonimmunocompromised controls. Each red square represents the OR for an individual study, with the size of the square proportional to the study's weight in the meta-analysis. Horizontal lines indicate the 95% CI for each study; wider intervals reflect lower precision due to smaller sample sizes or few positive cases. The vertical solid line at OR = 1 denotes the null value (no association). The black diamond at the bottom represents the pooled OR and its 95% CI. OR values to the right of the vertical line indicate increased odds of infection in immunocompromised patients (risk factor), while values to the left indicate a potential protective effect. The *x*-axis is plotted on a logarithmic scale.

**Figure 5:**
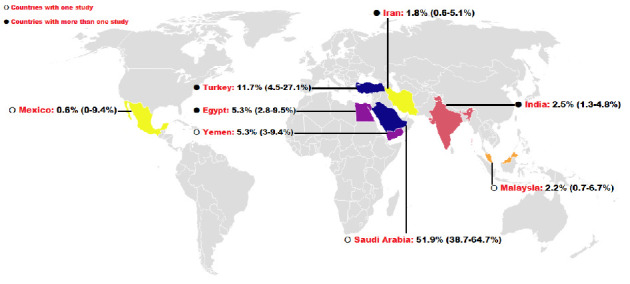
The distribution and prevalence of *C. cayetanensis* infection in immunocompromised patients by country.

The pooled prevalence of *C. cayetanensis* was 4% (95% CI: 2.1%–7.2%) in immunocompromised individuals (Figure 2), while it was only 1.2% (95% CI: 0.4%–3.4%) in nonimmunocompromised controls (Figure 3). This resulted in an OR of 5.4 (95% CI: 2.6–10.8, *p* < 0.001), indicating a significantly higher susceptibility of immunocompromised individuals to *C. cayetanensis* infections (Figure 4). The country-based prevalence of *C. cayetanensis* among immunocompromised patients is illustrated in Figure 5. The heterogeneity of prevalence data was assessed using the *I*^2^ statistic, which revealed substantial heterogeneity among cross-sectional (*I*^2^ = 86.8%, *p* < 0.001) and case–control (*I*^2^ = 57%, *p* < 0.05) studies.

### 3.3. Pooled Prevalence of *C. cayetanensis* Based on Patient Type

**Figure 6:**
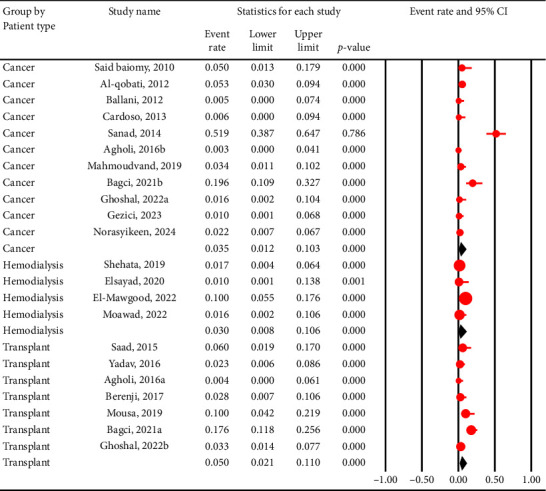
Forest plot showing the prevalence of *C. cayetanensis* infection among immunocompromised patients by patient types. Each horizontal black line represents the 95% confidence interval (CI) for the prevalence reported in an individual study. The red circles indicate the point estimate (event rate) for each study, with the size of the circle proportional to the study's weight in the meta-analysis. The vertical solid line at 0 represents the null value (no events), while the vertical dashed line represents the overall pooled prevalence estimated from the random-effects model. The diamonds summarize the pooled prevalence and its 95% CI in each group, with the center indicating the point estimate and the horizontal tips representing the CI limits.

Transplant recipients showed the highest pooled prevalence of *C. cayetanensis* infection among immunocompromised groups at 5% (95% CI: 2.1%–11%), followed by cancer patients at 3.5% (95% CI: 1.2%–10.3%) and individuals undergoing hemodialysis at 3% (95% CI: 0.8%–10.6%) (Figure 6).

### 3.4. Sensitivity Analysis and Subgroup-Based Prevalence

Removing individual studies did not significantly alter the pooled prevalence estimates (Supporting Figure 1). Furthermore, sensitivity analyses excluding studies reporting ORs showed no meaningful change in the overall OR, reinforcing the robustness of our findings. These results confirm that immunodeficiency is a significant and specific risk factor for *C. cayetanensis* infection, beyond the general susceptibility of immunocompromised patients to infections (Supporting Figure 2).  Table 2 outlines the pooled prevalence of *C. cayetanensis* infections by publication year, country, continent, WHO region, country income level, HDI value, and sample size (Supporting Figures 3–9).

### 3.5. Meta-Regression

**Figure 7:**
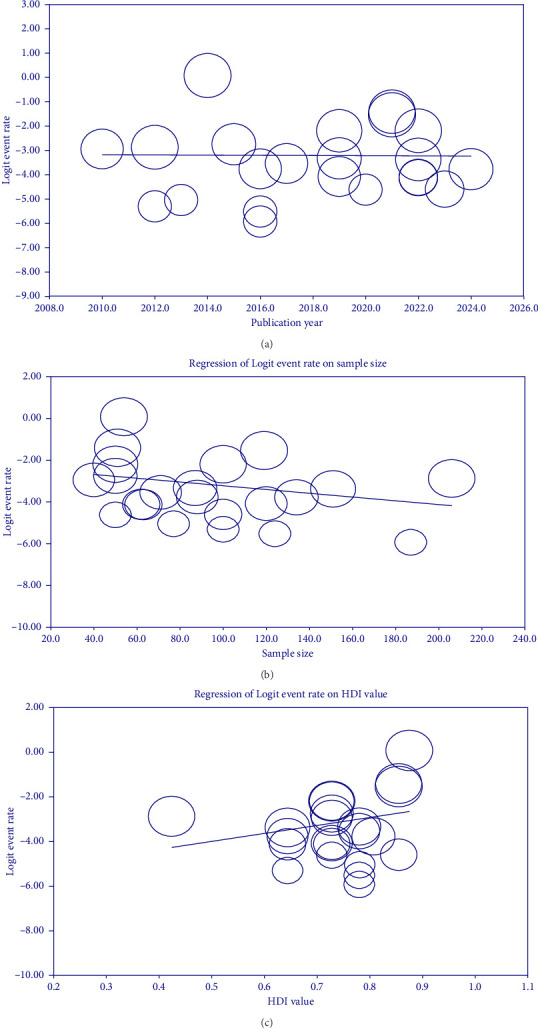
Bubble plots from meta-regression assessing the association between the logit-transformed prevalence of *C. cayetanensis* infection in immunocompromised patients and continuous moderators: (a) year of publication, (b) sample size of the included studies, and (c) Human Development Index (HDI) value of the study location. Each circle represents one study, with circle size proportional to the study's weight in the meta-analysis. The solid line shows the fitted meta-regression slope, and the shaded area represents the 95% confidence interval. No statistically significant associations were observed for any moderator (*p* > 0.05). The “logit event rate” corresponds to the logit-transformed proportion of infected individuals, calculated as the natural log of the odds (log[*p*/(1 − *p*)]) of infection in each study.

Our meta-regression analysis found no statistically significant association between the prevalence of *C. cayetanensis* infection in immunocompromised patients and quantitative variables like publication year (regression coefficient: −0.0039, *p* > 0.05), sample size (regression coefficient: −0.0090, *p* > 0.05), and HDI value (regression coefficient: 3.5684, *p* > 0.05). Therefore, the year of study, sample size, and HDI value did not account for variability in *C. cayetanensis* infection rates among immunocompromised patients (Figure 7).

### 3.6. Publication Bias

**Figure 8:**
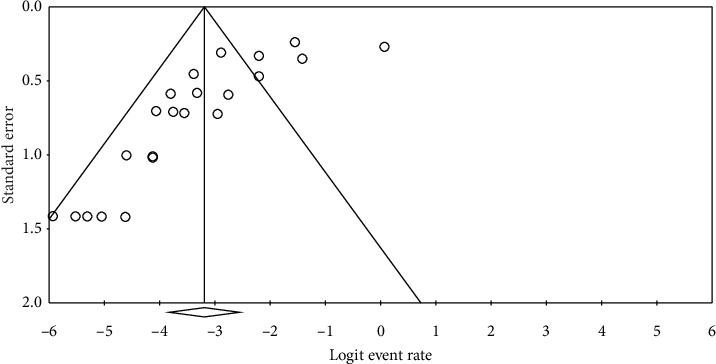
Funnel plot illustrating publication bias in the current systematic review and meta-analysis. The *x*-axis represents the logit event rate, while the *y*-axis displays the standard error. The plot's symmetry is an indicator of the potential publication bias, with smaller studies being dispersed at the bottom and larger studies toward the top. The triangles represent the expected distribution of studies under the assumption of no bias. Any asymmetry observed may suggest selective reporting or missing studies, potentially affecting the validity of the findings.

Visual inspection of the funnel plot and Egger's regression test indicated a degree of asymmetry, suggesting the presence of publication bias (*p* < 0.05) (Figure 8).

## 4. Discussion

The findings of the present study align with those of previous research, confirming that *C. cayetanensis* infection remains relatively uncommon, even among high-risk populations. The weighted prevalence of 4% (95% CI: 2.1%–7.2%) observed in immunocompromised patients (cancer, transplant, and hemodialysis) is comparable to the 3.9% (95% CI: 2.6%–5.4%) reported by Ramezanzadeh et al. [10] in HIV/AIDS patients. This suggests that various forms of immunosuppression may contribute similarly to susceptibility to *C. cayetanensis* infection.

In the current study, the pooled prevalence of *C. cayetanensis* was 4% (95% CI: 2.1%–7.2%) in immunocompromised individuals, compared to 1.2% (95% CI: 0.4%–3.4%) in control groups, indicating a notable disparity in infection rates. The calculated OR (5.4, 95% CI: 2.6–10.8, *p* < 0.001) further highlights a substantially increased risk of infection among immunocompromised individuals, suggesting that immune dysfunction significantly contributes to both the acquisition and prolonged persistence of *C. cayetanensis*. However, the relatively wide confidence interval observed for the OR indicates variability across the included studies and underscores the need for cautious interpretation. This heterogeneity may reflect differences in diagnostic methods, population characteristics, or regional exposures. Although immunocompromised individuals are generally more susceptible to infections, the risk of *C. cayetanensis* appears to be influenced by specific factors such as the degree of immune suppression, exposure to contaminated food or water, poor hygiene and nutrition, and limited access to timely diagnosis and care [31, 32]. These findings emphasize the importance of implementing targeted prevention strategies beyond routine infection control, particularly in endemic areas and among high-risk subgroups, through seasonal screening, improved food safety practices, and broader access to reliable diagnostic tools.

Subgroup analyses revealed that transplant recipients exhibited the highest pooled prevalence of *C. cayetanensis* infection among immunocompromised groups, followed by cancer patients and those undergoing hemodialysis. The higher pooled prevalence of *C. cayetanensis* infection observed in transplant recipients and cancer patients likely reflects the profound immunosuppression caused by their treatments, which can compromise mucosal barriers and reduce the host's ability to eliminate the pathogen [33]. However, pathogen-specific factors may also contribute to this susceptibility. For instance, the prolonged environmental viability of *C. cayetanensis* oocysts, the need for a minimal infectious dose, and its resilience in food and water sources may disproportionately affect individuals with impaired immune responses [2]. In contrast, the relatively lower prevalence in hemodialysis patients might be explained by differences in the nature of their immune dysfunction, more frequent clinical monitoring, and potentially lower environmental exposure. These findings suggest that both host-related and pathogen-specific characteristics must be considered when assessing infection risk in immunocompromised populations.

The sensitivity analysis confirmed the absence of influential outliers in studies reporting the prevalence and ORs of *C. cayetanensis* infection among immunocompromised individuals, indicating the robustness of the association between immunodeficiency and the likelihood of infection. The ORs specifically reflect the comparative odds of *C. cayetanensis* infection in immunocompromised patients versus immunocompetent controls. Although this increased risk should be more consistently observed in longitudinal and well-controlled studies, the overall pattern supports a meaningful association across diverse patient subgroups, suggesting that immunosuppression, regardless of its cause, may elevate susceptibility to infection by compromising the host's ability to clear the parasite.

In the current study, the visual inspection of the funnel plot and Egger's regression test suggests a degree of asymmetry, potentially suggesting publication bias. However, this visual pattern may reflect true heterogeneity rather than selective reporting, as most included studies (17 out of 22) reported low or zero prevalence (< 10%), and only a small number of studies showed high prevalence (> 10%). Meta-regression analyses were performed to explore whether publication year, sample size, or HDI of the study location explained variability in the reported prevalence of *C. cayetanensis* among immunocompromised patients. None of these moderators showed a statistically significant association with prevalence estimates (all *p* > 0.05). This suggests that the between-study heterogeneity is unlikely to be explained by these factors alone and may instead reflect differences in study design, diagnostic methods, or population characteristics. Although some funnel plot asymmetry was observed, it is more likely attributable to variations in study settings and sample sizes rather than systematic publication bias. Consequently, the pooled prevalence estimate appears reasonably robust; however, it should still be interpreted with caution.

The limited number of studies and regional disparities restricted definitive statistical conclusions, preventing the identification of a clear trend in *C. cayetanensis* prevalence among immunocompromised patients. Interestingly, pooled prevalence appeared higher in studies published after 2017 (4.8%) compared to those published before that year (2.9%). This increase raises concerns about potential shifts in environmental factors, food and water safety regulations, or improved diagnostic methodologies. Additionally, the geographic distribution of studies suggests that regional epidemiological factors might play a critical role.

Notably, the highest pooled prevalence of *C. cayetanensis* in immunocompromised patients was observed in Europe and the WHO EUR region (11.7%, 95% CI: 4.5%–27.1%), with Saudi Arabia [19] and Turkey [23, 26] reporting the highest national prevalence rates at 51.9% (95% CI: 38.7%–64.7%) and 11.7% (95% CI: 4.5%–27.1%), respectively. These findings highlight the need for further investigations into the environmental, public health, and sociocultural determinants contributing to these high infection rates. Factors such as water quality, agricultural practices, and food handling procedures likely play a significant role in facilitating transmission, as previously discussed in the literature [2, 32, 34].

Interestingly, countries with very high HDI value and high-income levels reported the highest pooled prevalence of *C. cayetanensis* infection among immunocompromised patients, despite having implemented advanced food safety regulations. This paradox may be explained by several factors. First, greater diagnostic capacity and surveillance systems in high-income countries likely lead to more accurate detection and reporting of infections. Second, immunocompromised patients in these settings may still be exposed to the parasite through the consumption of imported fresh produce or contaminated water sources, as *C. cayetanensis* oocysts are resilient to common disinfection methods and can survive in various environmental niches. Third, behavioral and dietary habits, such as increased consumption of raw fruits and vegetables, may contribute to ongoing exposure despite overall food safety improvements. Thus, while food safety measures reduce the general population's risk, immunosuppressed individuals remain vulnerable due to their impaired immune defenses and potential exposure routes that are not fully controlled by current safety practices.

A notable trend observed in this study was the inverse association between the sample size and prevalence estimates, with larger studies (sample size > 100) reporting a lower pooled prevalence of *C. cayetanensis* infection (3%) in immunocompromised individuals. While this highlights the importance of adequate sample size in improving the precision and reliability of epidemiological estimates, other contributing factors must also be considered. For example, studies with small sample sizes were often conducted in high-exposure settings or among subpopulations with severe immunosuppression, which could inflate prevalence figures. Additionally, variation in diagnostic methods, such as the use of PCR versus conventional microscopy can affect detection rates. Geographic differences, local sanitation conditions, and study design quality may also play substantial roles. Therefore, while sample size influences the stability of prevalence estimates, heterogeneity in methodological and contextual factors across studies necessitates cautious interpretation. These findings reinforce the need for standardized, multicenter studies using sensitive diagnostic tools to provide more generalizable and accurate prevalence data.

Despite the strengths of this meta-analysis, such as the inclusion of recent data, rigorous statistical methodologies, sensitivity analyses, meta-regression, and publication bias, the study has several limitations. The small number of included studies, limited geographic diversity, relatively small sample sizes, the restricted scope of variables included in the meta-regression analysis, and reliance on single studies/datasets for some analyses pose challenges to generalizability. Furthermore, the lack of age- and gender-specific data constrains the ability to explore demographic risk factors. Future research should focus on expanding geographical representation, increasing study sample sizes, and incorporating additional demographic variables to provide a more comprehensive understanding of *C. cayetanensis* epidemiology in immunocompromised populations.

## 5. Conclusion

This study highlights a significantly higher pooled prevalence of *C. cayetanensis* infection among immunocompromised individuals compared to immunocompetent controls, with over a fivefold increase in infection risk. Transplant recipients, cancer patients, and individuals undergoing hemodialysis were identified as particularly vulnerable subgroups. These findings underscore the opportunistic nature of *C. cayetanensis* and the need for heightened clinical awareness, especially in settings where foodborne exposure remains a concern. As the global population of immunosuppressed individuals continues to grow due to medical advances in oncology, transplantation, and chronic disease management, tailored prevention strategies, improved diagnostic access, and focused epidemiological surveillance are essential. Future research should further investigate host- and pathogen-related factors that drive infection risk and develop context-specific interventions to reduce the burden of cyclosporiasis in these high-risk populations.

## Figures and Tables

**Table 1:** Data from 19 articles/22 datasets on the prevalence of *C. cayetanensis* infection in immunocompromised patients and immunocompetent controls.

Author, year	Patient type	Time tested	Country	Cases		Controls		Study type	Methods
				Total no.	Prevalence (%)	Total no.	Prevalence (%)		
Baiomy et al., 2010 [5]	Cancer	UC	Egypt	40	5	20	0	C-C	MIC
Al-Qobati et al., 2012 [25]	Cancer	2011–2012	Yemen	206	5.3	—^a^	—^a^	C-S	MIC
Ballani et al., 2012 [27]	Cancer	UC	India	100	0	50	0	C-C	MIC
Jiménez-Cardoso et al., 2013 [16]	Cancer	2010–2011	Mexico	77	0	—	—	C-S	MIC, MOL
Sanad et al., 2014 [19]	Cancer	UC	Saudi Arabia	54	51.9	42	13.1	C-C	MIC
Saad et al., 2015 [8]	Transplant	2011–2013	Egypt	50	6	25	0	C-C	MIC
Yadav et al., 2016 [20]	Transplant	2011–2013	India	88	2.3	—	—	C-S	MIC
Agholi et al., 2016 [21]	Transplant	2009–2014	Iran	124	0	—	—	C-S	MIC, MOL
Agholi et al., 2016 [21]	Cancer	2009–2014	Iran	187	0	—	—	C-S	MIC, MOL
Berenji et al., 2017 [22]	Transplant	2015–2016	Iran	71	2.8	71	0	C-C	MIC
Mahmoudvand et al., 2019 [18]	Cancer	2017–2018	Iran	87	3.5	—	—	C-S	MIC
Ismail and Fadl, 2019 [28]	Transplant	2018–2019	Egypt	50	10	—	—	C-S	MIC
Shehata et al., 2019 [7]	Hemodialysis	2014–2016	Egypt	120	1.7	100	0	C-C	MIC
Elsayad et al., 2020 [30]	Hemodialysis	UC	Egypt	50	0	50	0	C-C	MIC
Bağci et al., 2021 [23]	Transplant	2016–2019	Turkey	119	17.6	—	—	C-S	MIC
Bağci et al., 2021 [23]	Cancer	2016–2019	Turkey	51	19.6	—	—	C-S	MIC
El-Mawgood et al., 2022 [17]	Hemodialysis	2020	Egypt	100	10	100	0	C-C	MIC
Ghoshal et al., 2022 [29]	Cancer	2007–2019	India	61	1.6	170	0	C-C	MIC
Ghoshal et al., 2022 [29]	Transplant	2007–2019	India	151	3.3	170	0	C-C	MIC
Moawad et al., 2022 [24]	Hemodialysis	2018–2019	Egypt	62	1.6	39	0	C-C	MIC
Gezici et al., 2023 [26]	Cancer	2017–2018	Turkey	100	1	100	0	C-C	MIC
Siti Farah Norasyikeen et al., 2024 [15]	Cancer	UC	Malaysia	134	2.2	17	0	C-C	MIC, MOL

*Note:* UC: unclear, C–C: case–control design, C-S: cross-sectional design, MIC: microscopic detection, and MOL: molecular detection.

^a^The dashed line (—) indicates that these studies did not have a control group.

**Table 2:** Subgroup analysis of *C. cayetanensis* infection in immunocompromised patients by publication year, continent, WHO region, country, HDI value, country income level, and sample size.

Subgroup variable	Prevalence % (95% CI)	Heterogeneity (Q)	df (Q)	*I* ^2^ (%)	*p* value
Publication year					
2010–2017	2.9 (0.8–10.2)	103.7	9	91.3	*p* < 0.05
2018–2024	4.8 (2.5–8.8)	52.1	11	78.9	*p* < 0.05
Continent					
Africa	5.3 (2.8–9.5)	10.9	6	45.3	*p* > 0.05
Asia	2.8 (0.9–8.6)	121.1	10	91.7	*p* < 0.05
Europe	11.7 (4.5–27.1)	9.2	2	78.3	*p* < 0.05
Central America	0.6 (0–9.4)	0	0	0	*p* > 0.05
WHO region					
AMR	0.6 (0–9.4)	0	0	0	*p* > 0.05
EMR	4.2 (1.7–9.9)	109	12	89	*p* < 0.05
EUR	11.7 (4.5–27.1)	9.2	2	78.3	*p* < 0.05
SEAR	2.5 (1.3–4.8)	1.9	3	0	*p* > 0.05
WPR	2.2 (0.7–6.7)	0	0	0	*p* > 0.05
Country					
Egypt	5.3 (2.8–9.5)	10.9	6	45.3	*p* > 0.05
India	2.5 (1.3–4.8)	1.9	3	0	*p* > 0.05
Iran	1.8 (0.6–5.1)	4.5	3	33.1	*p* > 0.05
Malaysia	2.2 (0.7–6.7)	0	0	0	*p* > 0.05
Mexico	0.6 (0–9.4)	0	0	0	*p* > 0.05
Saudi Arabia	51.9 (38.7–64.7)	0	0	0	*p* > 0.05
Turkey	11.7 (4.5–27.1)	9.2	2	78.3	*p* < 0.05
Yemen	5.3 (3–9.4)	0	0	0	*p* > 0.05
HDI value^∗^					
High	3.4 (1.8–6.1)	23.9	11	54	*p* < 0.05
Low	5.3 (3–9.4)	0	0	0	*p* > 0.05
Medium	2.5 (1.3–4.8)	1.9	3	0	*p* > 0.05
Very high	11.6 (3.7–31.1)	55.1	4	92.7	*p* < 0.05
Country income level					
High	51.9 (38.7–64.7)	0	0	0	*p* > 0.05
Low	5.3 (0.3–9.4)	0	0	0	*p* > 0.05
Lower middle	3.9 (2.3–6.6)	19.2	10	47.9	*p* > 0.05
Upper middle	3.1 (1.1–8)	49.1	8	83.7	*p* < 0.05
Sample size					
≤ 100	4.4 (1.9–9.8)	108.9	14	87.1	*p* < 0.05
> 100	3 (1.1–7.8)	41.1	6	85.4	*p* < 0.05

*Note:* World Health Organization (WHO) region. Df (Q): This column represents the degrees of freedom associated with the heterogeneity test (Q statistic). It indicates the number of independent comparisons used to assess the heterogeneity among studies. The value provides context for interpreting the Q statistic and the *I*^2^ metric, helping determine if the variability between studies is greater than what would be expected by chance alone. *I*^2^ for heterogeneity: The *I*^2^ statistic quantifies the percentage of total variation across studies due to heterogeneity rather than chance. It provides a clearer understanding of the degree of consistency among study results, where values closer to 0% suggest low heterogeneity and values closer to 100% indicate high heterogeneity. In this study, heterogeneity was measured using the *I*^2^ statistic, with values exceeding 50% indicating substantial heterogeneity. Threshold for interpretation: The threshold for interpreting heterogeneity, such as a p value threshold (typically < 0.05), helps determine whether the heterogeneity observed is statistically significant.

Abbreviation: HDI, Human Development Index.

^∗^HDI values above 0.800 are classified as very high, those between 0.700 and 0.799 as high, from 0.550 to 0.699 as medium, and below 0.550 as low.

## Data Availability

The data that support the findings of this study are available in the supporting information of this article.

## Supporting Information

Supporting Information 1 Figure S1: The sensitivity analysis to check for the influence of any single study on the pooled estimates.

Supporting Information 2 Figure S2: The sensitivity analysis to check for the influence of any single study on the weighted ORs.

Supporting Information 3 Figure S3: The pooled prevalence of *C. cayetanensis* infection in immunocompromised patients based on publication years. Red indicates the prevalence from each study, while black shows the overall weighted prevalence.

Supporting Information 4 Figure S4: The pooled prevalence of *C. cayetanensis* infection in immunocompromised patients based on continents. Red indicates the prevalence from each study, while black shows the overall weighted prevalence.

Supporting Information 5 Figure S5: The pooled prevalence of *C. cayetanensis* infection in immunocompromised patients based on WHO regions. Red indicates the prevalence from each study, while black shows the overall weighted prevalence.

Supporting Information 6 Figure S6: The pooled prevalence of *C. cayetanensis* infection in immunocompromised patients based on countries. Red indicates the prevalence from each study, while black shows the overall weighted prevalence.

Supporting Information 7 Figure S7: The pooled prevalence of *C. cayetanensis* infection in immunocompromised patients based on HDI values. Red indicates the prevalence from each study, while black shows the overall weighted prevalence.

Supporting Information 8 Figure S8: The pooled prevalence of *C. cayetanensis* infection in immunocompromised patients based on country-income levels. Red indicates the prevalence from each study, while black shows the overall weighted prevalence.

Supporting Information 9 Figure S9: The pooled prevalence of *C. cayetanensis* infection in immunocompromised patients based on sample sizes. Red indicates the prevalence from each study, while black shows the overall weighted prevalence.

Supporting Information 10 Table S1: JBI critical appraisal checklist applied for included studies.#
