# Supplementary material for: Prevalence and Associated Risk Factors of Cyclospora cayetanensis in Immunocompromised Patients: A Systematic Review and Meta-Analysis
Source: Can J Infect Dis Med Microbiol. 2025 Aug 28;2025:8837624. doi: 10.1155/cjid/8837624 (PMC12411048; doi:10.1155/cjid/8837624)
Supplement: Supporting Information 1 — Figure S1: The sensitivity analysis to check for the influence of any single study on the pooled estimates. [file 8837624.f1.docx]

**Supplementary Fig. 1.** The sensitivity analysis to check for the influence of any single study on the pooled estimates.
