# Supplementary material for: Prevalence and Associated Risk Factors of Cyclospora cayetanensis in Immunocompromised Patients: A Systematic Review and Meta-Analysis
Source: Can J Infect Dis Med Microbiol. 2025 Aug 28;2025:8837624. doi: 10.1155/cjid/8837624 (PMC12411048; doi:10.1155/cjid/8837624)
Supplement: Supporting Information 6 — Figure S6: The pooled prevalence of C. cayetanensis infection in immunocompromised patients based on countries. Red indicates the prevalence from each study, while black shows the overall weighted prevalence. [file 8837624.f6.docx]

**Supplementary Fig. 6.** The pooled prevalence of *C. cayetanensis* infection in immunocompromised patients based on countries. Red indicates the prevalence from each study, while black shows the overall weighted prevalence.
